# *In vitro* Assessment of the DNA Damage Response in Dental Mesenchymal Stromal Cells Following Low Dose X-ray Exposure

**DOI:** 10.3389/fpubh.2021.584484

**Published:** 2021-02-15

**Authors:** Niels Belmans, Liese Gilles, Jonas Welkenhuysen, Randy Vermeesen, Bjorn Baselet, Benjamin Salmon, Sarah Baatout, Reinhilde Jacobs, Stéphane Lucas, Ivo Lambrichts, Marjan Moreels

**Affiliations:** ^1^Morphology Group, Biomedical Research Institute (BIOMED), Hasselt University, Diepenbeek, Belgium; ^2^Belgian Nuclear Research Centre, Institute for Environment, Health and Safety, Radiobiology Unit, Mol, Belgium; ^3^Environmental Risk and Health Unit, Flemish Institute for Technological Research (VITO), Mol, Belgium; ^4^PXL Tech, PXL, Diepenbeek, Belgium; ^5^Université de Paris, Orofacial Pathologies, Imaging and Biotherapies UR2496 Lab, Montrouge, France; ^6^Dental Medicine Department, AP-HP, Bretonneau hospital, Paris, France; ^7^Oral and Maxillofacial Surgery, Dentomaxillofacial Imaging Center, Department of Imaging and Pathology, OMFS-IMPATH Research Group, and University Hospitals, Katholieke Universiteit Leuven, Leuven, Belgium; ^8^Department Dental Medicine, Karolinska Institutet, Huddinge, Sweden; ^9^Laboratory of Analysis by Nuclear Reaction (LARN/PMR), Namur Research Institute for Life Sciences, University of Namur, Namur, Belgium

**Keywords:** dental stem cell, DNA damage response, DNA double strand break, low dose radiation exposure, cell cycle, cellular senescence

## Abstract

Stem cells contained within the dental mesenchymal stromal cell (MSC) population are crucial for tissue homeostasis. Assuring their genomic stability is therefore essential. Exposure of stem cells to ionizing radiation (IR) is potentially detrimental for normal tissue homeostasis. Although it has been established that exposure to high doses of ionizing radiation (IR) has severe adverse effects on MSCs, knowledge about the impact of low doses of IR is lacking. Here we investigated the effect of low doses of X-irradiation with medical imaging beam settings (<0.1 Gray; 900 mGray per hour), *in vitro*, on pediatric dental mesenchymal stromal cells containing dental pulp stem cells from deciduous teeth, dental follicle progenitor cells and stem cells from the apical papilla. DNA double strand break (DSB) formation and repair kinetics were monitored by immunocytochemistry of γH2AX and 53BP1 as well as cell cycle progression by flow cytometry and cellular senescence by senescence-associated β-galactosidase assay and ELISA. Increased DNA DSB repair foci, after exposure to low doses of X-rays, were measured as early as 30 min post-irradiation. The number of DSBs returned to baseline levels 24 h after irradiation. Cell cycle analysis revealed marginal effects of IR on cell cycle progression, although a slight G_2_/M phase arrest was seen in dental pulp stromal cells from deciduous teeth 72 h after irradiation. Despite this cell cycle arrest, no radiation-induced senescence was observed. In conclusion, low X-ray IR doses (< 0.1 Gray; 900 mGray per hour), were able to induce significant increases in the number of DNA DSBs repair foci, but cell cycle progression seems to be minimally affected. This highlights the need for more detailed and extensive studies on the effects of exposure to low IR doses on different mesenchymal stromal cells.

## Introduction

Stem cells contained within the dental mesenchymal stromal cell (MSCs) population are of paramount importance for tissue homeostasis which are potentially important targets of ionizing radiation (IR) exposure. They can accumulate genotoxic damage following IR exposure, which is either repaired efficiently, or they can accumulate irreversible damage. This irreversible damage can trigger apoptosis or senescence, and misrepaired or unrepaired DNA damage can persist and could potentially lead to malignant transformation of the stem cells (1). Changes in the functionality of MSCs could therefore be considered as a predictive indicator for future health hazards (2, 3).

In 2000, Gronthos et al. identified and isolated odontogenic progenitor cells from the dental pulp from adult patients (4). These cells were dubbed dental pulp stem cells (DPSCs). In the following years, several more types of dental stem cells were described, such as the dental follicle stem cells (DFSCs), stem cells from the apical papilla (SCAPs), pulp stem cells from human exfoliated deciduous teeth (SHEDs), and periodontal ligament stem cells (PDLSCs) (5–8). However, the International Society for Cellular Therapy has prompt to define the isolation of mesenchymal stem cells as non-clonal cultures of stromal cells containing stem cells with different multipotent properties, committed progenitors, and differentiated cells (9–11). An overview of these cells and their potential use in dentistry is given by Bansal and Jain (12).

Today, one of the greatest challenges in radiation protection is unraveling the detrimental effects of exposure to low doses of IR. This is important because people are exposed to low dose IR on a daily basis, either from natural sources, or from man-made sources, such as medical diagnostics (13). Although there are epidemiological data on exposure to doses higher than 100 mGy (e.g., from atomic bomb survivors, medically and occupationally exposed populations and environmentally exposed groups), no conclusive data exists on exposure to low doses of IR (14). Currently, risk estimation for low dose exposure is based on linear extrapolation from these high dose data. This model is the famous linear-no-threshold (LNT) model (15–17). The LNT model assumes that there is a linear relationship between IR dose and the excessive cancer risk. When applying the LNT model, the following is assumed: (1) that there is a linear relationship between IR dose and the amount of radiation-induced DNA double strand breaks (DSB), (2) that each DNA DSB has the probability of inducing cellular transformations, and (3) that each transformation has the same probability of resulting in carcinogenesis (18). However, in the low dose range (<100 mGy), other phenomena than a linear response can occur. There is evidence that low doses of IR could have beneficial effects, such as hormesis and adaptive responses (19, 20). Hormesis occurs when exposure to low IR doses produces a favorable effect, whereas high IR doses result in detrimental effects (21). Adaptive responses occur when a very low dose, or priming dose, stimulates cells which results in increased resistance to a second, larger dose of the same trigger at a later time point. This could include the activation of genes associated with DNA damage repair, stress scavenging, cell cycle control and apoptosis (19, 20).

DNA DSBs are the most crucial DNA lesions that are associated with increased cancer risk and IR exposure. If not repaired correctly, DSBs can cause genomic instability, mutations, chromosome aberrations and translocations, and cell death (22–25). To protect the DNA against these types of damage, eukaryotes have developed the DNA damage response (DDR) (24, 25). In short, cellular responses to IR-induced DNA DSBs are triggered by the activation of the ataxia telangiectasia mutated (ATM) kinase. The phosphorylation of histone H2AX on serine 139 (γH2AX) in the vicinity of the DNA DSB is one of the earliest ATM-dependent responses, although other kinases are also capable of phosphorylating histone H2AX on serine 139 (23, 26, 27). γH2AX forms so called DNA damage foci in the nucleus, or in the case of IR-induced DNA damage “IR-induced foci” (IRIF). In general, IRIF are distinct sub-nuclear structures to which the DDR proteins re-localize. After phosphorylation, γH2AX initiates a signaling cascade leading to the recruitment of multiple DDR proteins, including tumor suppressor p53-binding protein 1 (53BP1) (22, 24, 28, 29).

53BP1 is a known DNA DSB sensor and a mediator and effector in the DDR to DSBs (24, 30, 31). Similar to γH2AX, 53BP1 has several functions in the DDR, such as recruitment of DSB repair proteins, checkpoint signaling, determining the DSB repair pathway and synapsis of distal DNA ends during non-homologous end-joining (reviewed in Panier and Boulton) (30).

Evidence shows that both γH2AX and 53BP1 show a quantitative relationship between the number of foci and the number of DNA DSBs (24, 29, 32, 33). Although γH2AX is a powerful tool to monitor DNA DSBs, artifacts do occur even in the absence of DSBs (25). Both γH2AX and 53BP1 foci can be visualized using immunofluorescence microscopy and are detectable within minutes following exposure to IR (29, 34). Therefore, using an immunostaining protocol for simultaneous detection of γH2AX and 53BP1 allows for better estimation of the amount of DSBs present and it reduces the impact of artifacts, since it is known that γH2AX and 53BP1 co-localize in IRIF (24, 35, 36).

DNA DSB could be efficiently repaired by the DDR, although misrepair can occur. However, DNA DSBs could persist. This could lead to cell cycle arrest, premature cellular senescence, or apoptosis. As part of the DDR, cells halt their passage through the cell cycle, allowing DDR proteins to repair DNA damage. If this damage persists, the cell cycle could be irreversibly arrested. This cell cycle arrest can occur in all phases of the cell cycle, but it was found that most cells are most sensitive to IR-induced DNA damage in the G_2_/M phase (37–39). Cellular senescence is a state of irreversible growth arrest. This growth arrest occurs in the G_1_ phase of the cell cycle, therefore cellular senescence is linked with changes in cell cycle progression. A hallmark of senescent cells is the increased β-galactosidase activity in comparison to normal cells. This can be detected by the so-called X-gal assay, which is considered as the gold standard for senescence testing (40, 41). Senescent cells also display a senescence-associated secretory phenotype (SASP), which consists of several chemokines, cytokines, and regulatory factors. Some of these SASP factors are linked with IR exposure, such as IL-6, IL-8, IGFBP-2, and IGFBP-3 (42, 43). IL-6 and IL-8 interact with their surface receptors, which initiates several intracellular pathways. Besides that, they can both induce or reinforce senescence in damaged cells in a paracrine/autocrine manner (42, 43). IGFBP-2 and IGFBP-3 interact with insulin-like growth factor (IGF). They sequester IGF so it cannot bind to its receptor, which eventually leads to inhibition of cell proliferation (44). It is known that premature cellular senescence can be caused by several stresses, such as (persisting) DNA damage or reactive oxygen species (45). It has been reported before that exposure to (high) IR doses can cause premature cellular senescence. This was observed both in mesenchymal stem cells and normal tissue cells (46–51). For low doses of IR, data is more scarce (3, 52). Besides senescence, quiescence is also an important process in stem cells. Quiescence is characterized by a cell cycle arrest in the G_0_ phase. This phase is similar to the G_1_ phase, however cells do not progress into the S phase. Unlike senescence, quiescence is a state of reversible growth arrest. Quiescence occurs in cells that require a strict proliferation regime, such as stem cells. It allows stem cells to assure genomic integrity until they are needed for tissue repair, which is when they are stimulated to reprise the normal cell cycle (53). Evidence on the effects of IR on quiescence in mesenchymal stem cells are scarce (54, 55). Finally, cells can undergo apoptosis or programmed cell death. Like premature cellular senescence, it is a response to extensive cellular stress and mostly occurs when DNA damage repair is slow and/or incomplete (56).

The aim of this study is to investigate the effects of low dose X-ray exposure with medical imaging beam settings (< 100 mGy; 900 mGy/h) on SHED, DFSCs, and SCAPs extracted from pediatric patients. DNA DSB formation and repair, cell cycle progression, cellular quiescence, and cellular senescence were monitored at several time points after exposure. Our data evidences that, although low doses of IR induce significant amounts of DNA DSBs, DNA damage is effectively repaired and does not affect cell cycle progression, nor induces premature cellular senescence in dental mesenchymal stromal cells.

## Materials and Methods

### Ethical Approval for the Use of Donor-Derived Dental Mesenchymal Stromal Cells

The cells were gifted by Prof. Benjamin Salmon (Dental Medicine Department of the Bretonneau Hospital (Paris, France). All experiments and methods were performed in accordance with relevant guidelines and regulations. All experimental protocols were approved by a named institutional/licensing committee. Ethical approval was obtained at the Comité d'Evaluation de l'Ethique des projets de Reserche Biomédicale Paris Nord, N°16-021 in France.

### Culturing Dental Stem Cells

Three types of dental mesenchymal stromal cells from different pediatric donors were used in this experiment: dental pulp stem cells from deciduous teeth (SHED−3 donors), dental follicle stem cells (DFSC−2 donors), and stem cells from the apical papilla (SCAP−3 donors). These cells were extracted from teeth as previously described (4, 5, 8). Yet, criteria recommended by The International Society for Cellular Therapy were not systematically verified and our findings rely on the extensive expertise of Prof. Benjamin Salmon (57–61) First, teeth were decontaminated using a povidone-iodine solution. Second, they were sectioned and exposed pulp tissues were collected. Third, their tissues were enzymatically digested using a type I collagenase and dispase solution. Finally, the cells were ready to be cultured. After extraction, the cells were seeded at a density of 10^4^ cells per cm^2^. They were grown in Dulbecco's Modified Eagle Medium (DMEM) containing 1 g/l D-glucose, GlutaMAX^TM^ and 10% fetal bovine serum (FBS) at 37°C with 5% CO_2_ in a humidified incubator. The medium was refreshed every 2–3 days. At 70–80% confluence the cells were passaged and seeded again at 10^4^ cells per cm^2^, or frozen in liquid nitrogen for later use. To be sure that the stem cells keep their phenotype, all stem cells were used between passage 1 and 5. Once enough cells were obtained they were seeded either into 8-chamber Labtek II slides at 2 x 10^4^ cells per well or in 24-well plates at 4 × 10^4^ cells per well (Greiner Bio-One, Frickenhausen, Germany) 24 h before irradiation. Six wells in each Labtek were used, resulting in six technical replicates. Each Labtek represented one time point per dose. In the 24-well plates cells were seeded in triplicates. For each cell type (SHED, SCAP, or DFSC), cells from three donor children were used (*N* = 3). For each experiment, the cell type from one donor child was considered as being one biological replicate (Table 1).

**Table 1:** Overview of dental stromal cell donors.

	**Age**	**Gender**
Donor 1	12	Male
Donor 2	11	Female
Donor 3	8	Female

### X-irradiation Conditions

Samples were irradiated at the Belgian Nuclear Research Centre (SCK CEN) with a XStrahl 320 kV Generator (Surrey, UK). In this experimental design, it is of importance to mimic commercially available Cone Beam Computed Tomography devices as closely as possible. To this end X-rays with RQR9 beam settings were used since it can be used to simulate entrance beams used in diagnostic radiology. The X-ray tube used a tube voltage of 120 kiloVolt and a current of 1.8 milliAmpere. The X-ray beam was filtered by 2.9 mm of aluminum. Using these parameters low doses and lower dose rates can be achieved which allows the simulation of diagnostic examinations. Using a dose rate of 900 mGy per hour the samples were irradiated with doses of 100 ± 1.9, 50 ± 0.9, 20 ± 0.38, 10 ± 0.19, and 5 ± 0.10 mGy.

### Immunocytochemical Staining for γH2AX and 53BP1

At specific time points after irradiation exposure (0.5, 1, 4, and 24 h) the culture medium was removed from the Labteks^TM^ (Nunc^TM^, ThermoFisher Scientific, Waltham, MA, USA). Then the cells were washed twice using 1x phosphate buffered saline (PBS). After washing, they were fixed in 2% paraformaldehyde (PFA) in 1x PBS for at least 15 min at room temperature (RT). Next the PFA was removed and the cells were washed twice with 1x PBS.

Fixed stem cells were double stained for γH2AX and 53BP1, both markers for DNA DSBs. The 1x PBS was removed and then the cells were permeabilized by incubating them in 0.25% Triton X-100 in 1x PBS for 3 min at RT. Then the cells were washed three times in 1x PBS on a rocking platform. Next the cells were blocked in pre-immunized goat serum (PIG). The PIG was diluted (1:5) in Tris-HCl – NaCl blocking buffer (50 mM Tris-HCl, 150 mM NaCl, 0.1% Tween 20, 0.5% blocking reagent (FP1012, Perkin Elmer) (TNB). The cells were blocked for 1 h at RT on a rocking platform, during which the primary antibody solution was prepared. Primary antibodies were diluted in TNB, the mouse anti-human γH2AX monoclonal antibody (05-636, Millipore, Massachusetts, USA) was diluted 1:300 and the rabbit anti-human 53BP1 polyclonal antibody (NB100-304, Novus Biological, Abingdon, UK) was diluted 1:1,000. After blocking, the cells were incubated with the primary antibody solution for 1 h at 37°C on a rocking platform. After incubation, the cells were washed three times using 1x PBS. Next the secondary antibody solution was prepared. An Alexa fluor 488-labeled goat anti-mouse antibody (A11001, Life Technologies, Oregon, USA) and an Alexa fluor 568-labeled goat anti-rabbit antibody (A11011, Life Technologies, Oregon, USA) were diluted 1:300 and 1:1,000 in TNB, respectively. The cells were incubated with the secondary antibody solution for another hour at 37°C on a rocking platform. After this final incubation step, the cells were washed twice using 1x PBS. Next the chambers were removed from the Labteks®. Then the samples were mounted using Prolong® Diamond Antifade Mountant with 4',6-diamidino-2-phenylindole (DAPI) (P36962, Molecular Probes^TM^ by Life Technologies, Oregon, USA) as nuclear counter stain. After mounting, the samples were stored at −20°C until imaging.

Images were acquired with a Nikon Eclipse Ti fluorescence microscope using a 40x dry objective (Nikon, Tokyo, Japan). Per technical replicate (*n* = 6 = number of chamber of a Labtek^TM^ used) at least 250 cells were counted. Afterwards, the images were analyzed using Fiji open source software (62). Fiji allows for analysis of each separate nucleus based on the DAPI signal. Within each nucleus, the intensity signal for the Alexa fluorophores were analyzed, after which the number of co-localized γH2AX and 53BP1 foci per nucleus were determined in a fully automated manner by using the Cellblocks tool (63).

### Cell Cycle Analysis

Cell cycle analysis was performed 1, 4, 24, and 72 h after X-irradiation as described before (46). In short, dental stem cells were treated with 10 μM of BrdU for 1 h. Afterwards, the cells were fixed with ice-cold 70% ethanol and stored for a minimum of 24 h. Next, the cells were permeabilized and stained with rat anti-BrdU antibody, diluted 1 in 600 (AB6326, Abcam, Cambridge, UK). They were also stained with 10 μg/ml of a 7-amino-actinomycin D (7-AAD) solution (Sigma-Aldrich). Samples were analyzed on a BD Accuri C6 flow cytometer, with a maximum flow speed of 300 events per second. At least 20,000 cells were counted per sample.

### Quiescence Assay

G_0_ phase cells were identified 1, 4, 24, and 72 h after X-irradiation using a quiescence assay. Dental stem cells were fixed with ice-cold 70% ethanol following X-irradiation. Next, the cells were washed twice with 5% FBS (Gibco, Massachusetts, USA) and 0.25% Triton X-100 (Sigma-Aldrich, Missouri, USA) in 1x PBS (PFT). Next, the cells were stained with 10 μg/ml 7-AAD (A9400-1MG, Sigma-Aldrich, Missouri, USA) and 0.4 μg/ml pyronin Y (83200-5G, Sigma-Aldrich, Missouri, USA) for 20 min at RT. Samples were analyzed on a BD Accuri C6 flow cytometer, with a maximum flow speed of 300 events per second. At least 20,000 cells were counted per sample (64).

### β-galactosidase Assay

Senescence was assessed 1, 3, 7, and 14 days after X-irradiation using the senescence-associated β-galactosidase assay (ab65351, Abcam, Cambridge, UK) (41). Cells were fixed for 15 min at RT using the fixative solution provided with the kit. Next the cells were washed twice with 1x PBS. Then, the cells were stained with 1 mg/ml X-gal solution at 37°C for 18 h. Afterwards, the staining was stopped by adding 1 M Na_2_CO_3_. Next, the cells were incubated for 1 h at RT with a Giemsa dye, diluted 1:50 in 0.2 M acetate buffer (pH = 3.36). Finally, the cells were washed twice with Milli-Q water and allowed to air dry. At least 300 cells per sample were analyzed using a Nikon Eclipse Ti bright field microscope using a 5x dry objective (Nikon, Tokyo, Japan).

### Enzyme-Linked Immunosorbent Assay: IL-6, IL-8, IGFBP-2, and IGFBP-3

For senescence assays on cytokine secretion, supernatant was collected 1, 3, 7, and 14 days following irradiation. Dental stem cells were grown in 12-well plates. One milliliter of medium was collected at each time point. After the supernatant was collected, the cells were collected and counted by microscope. Supernatant samples were used for the ELISA for the detection of IL-6, IL-8, IGFBP-2, and IGFBP-3. ELISA was performed following manufacturer's instructions (DY206, DY208, DY674, and DY675, R&D Systems). Briefly, 96-well plates were coated overnight with a capture antibody. Next, the wells were washed with washing buffer. Blocking buffer was added and the plate was incubated for 1 h at RT. After blocking, the plate was washed one with washing buffer. Next, the supernatant was added and incubated for 2 h at RT. The plate was washed again, after which the detection antibodies were added and the plate was incubated for 2 h at RT. Next, the plate was washed with washing buffer and a streptavidin-horse radish peroxidase-labeled antibody was added and the plate was incubated for 20 min in the dark at RT. Then, the plate was washed with washing buffer. Next, the substrate solution was added and the plate was incubated for 20 min in the dark at RT. Afterwards, 2 M H_2_SO_4_ was added to stop the substrate reaction. The optical density was measured at 450 nm and 570 nm using a spectrophotometer (CLARIOstar, BMG Labtech, Offenburg, Germany).

### Statistical Analysis

Statistical analyses were performed using GraphPad Prism 8.0.0 (GraphPad Software Inc., San Diego, USA). Graphs show mean ± standard error of the mean. Two-way analysis of variance followed by *post-hoc* tests was performed to analyse both time- and dose-dependent effects. *P* < 0.05 was considered statistically significant.

## Results

### Exposure to Low Doses of X-rays Induces DSBs and Activates the DNA Damage Response in Dental Mesenchymal Stromal Cells

**Figure 1:**
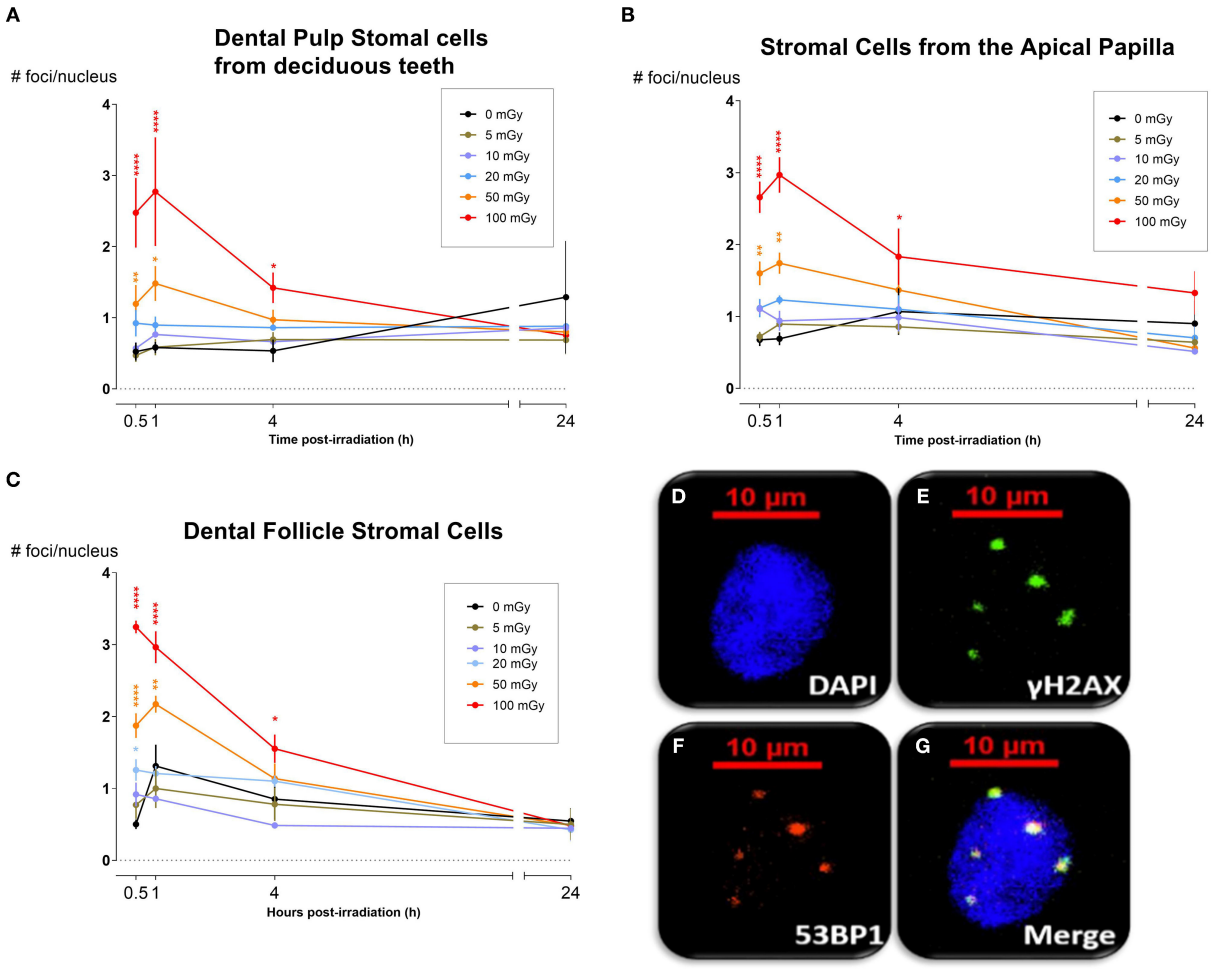
DNA double strand break formation and repair kinetics. **(A)** Dental pulp stromal cells from deciduous teeth show a significantly increased number of DNA double strand breaks following irradiation with 50 and 100 mGy 30 min and 1 h after radiation exposure. **(B)** The number of co-localized foci, observed in stromal cells from the apical papilla after exposure to 100 mGy, was significantly increased compared to 0 mGy 30 min, 1 and 4 h after irradiation (*P* < 0.0001, *P* < 0.0001, *P* = 0.0267, respectively). 50 mGy irradiated samples showed more foci 30 min and 1 h p.i (*P* = 0.0018, *P* = 0.0004, respectively). **(C)** In dental follicle stromal cells, more foci were observed 30 min, 1 and 4 h after exposure to 100 mGy (*P* < 0.0001, *P* < 0.0001, *P* = 0.0374, respectively). Thirty minutes and one hour after exposure to 50 mGy and 30 min after exposure to 20 mGy the amount of co-localized foci was increased as well in DFSC (*P* < 0.0001, *P* = 0.0015, *P* = 0.0030, respectively). The number of foci returns to control levels 24 h after irradiation. **(D–G)** Representative image from stromal cells from the apical papilla taken 60 min after irradiation with 100 mGy. The nucleus **(D)** shows five clear γH2AX **(E)** and 53BP1 **(F)** foci, which co-localize **(G)**. *P ≤ 0.05; **P ≤ 0.001; ****P < 0.0001.

DNA DSB formation and repair kinetics were monitored in dental mesenchymal stromal cells (SHED, DFSC, and SCAP), that were isolated from pediatric donors, by microscopic analysis of co-localized γH2AX and 53BP1 foci (*N* = 3). The number of co-localized foci was determined 30 min, 1, 4, and 24 h after X-irradiation with 0, 5, 10, 20, 50, and 100 mGy (dose rate: 900 mGy/h; Figure 1). The number of co-localized foci increased with increasing radiation dose. Typically, the peak response was seen between 30 and 60 min post-irradiation. After this period, the number of foci decreased until baseline levels were reached 24 h after exposure. More specifically, in SHED, exposure to 100 mGy induced significantly more co-localized foci 30 min and 1 h after irradiation compared to control cells (0 mGy) (*P* < 0.0001). A dose of 50 mGy also resulted in more co-localized foci 1 h after irradiation compared to 0 mGy (*P* = 0.0303). In the SCAPs, the number of co-localized foci, observed after exposure to 100 mGy, was significantly increased compared to 0 mGy 30 min, 1 and 4 h after irradiation (*P* < 0.0001, *P* < 0.0001, *P* = 0.0267, respectively). Furthermore, compared to control samples, 50 mGy irradiated samples showed more foci 30 min and 1 h p.i (*P* = 0.0018, *P* = 0.0004, respectively) and 20 mGy irradiated samples showed more foci 1 h after irradiation (*P* = 0.0416). In DFSC, more γH2AX and 53BP1 co-localized foci were observed 30 min, 1 h and 4 h after exposure to 100 mGy (*P* < 0.0001, *P* < 0.0001, *P* = 0.0374, respectively). Thirty minutes and one hour after exposure to 50 mGy and 30 min after exposure to 20 mGy the amount of co-localized foci was increased as well in DFSC (*P* < 0.0001, *P* = 0.0015, *P* = 0.0030, respectively). Furthermore, linear regression plots show a linear dose response 30 min, 1 h and 4 h after irradiation. Moreover, the slope decreased over time returning to a constant basal response 24 h after irradiation. Our linear regression analysis also resulted in a slope of about 0.020 DNA DSBs per mGy (Table 2). No difference in radiation sensitivity was observed between the different stromal cell types.

**Table 2:** Linear dose response relationship of co-localized γH2AX and 53BP1 foci in dental stromal cells.

**Cell type**	**Time after irradiation**	**Slope (foci/mGy)**	***R*^2^-value**	***P-*value**
Dental pulp stromal cells from deciduous teeth (SHEDs)	30 min	0.020	0.97	0.0003
	1 h	0.022	0.99	<0.0001
	4 h	0.008	0.96	0.0005
	24 h	−0.002	0.18	0.40
Dental follicle stromal cells (DFSCs)	30 min	0.026	0.99	<0.0001
	1 h	0.020	0.91	0.003
	4 h	0.008	0.75	0.025
	24 h	−0.0001	0.013	0.83
Stromal cells from the apical papilla (SCAPs)	30 min	0.019	0.98	0.0002
	1 h	0.022	0.99	<0.0001
	4 h	0.009	0.94	0.0012
	24 h	0.005	0.47	0.13

### Cell Cycle Progression Is Not Influenced by Low Doses of X-rays in Dental Mesenchymal Stromal Cells

**Figure 2:**
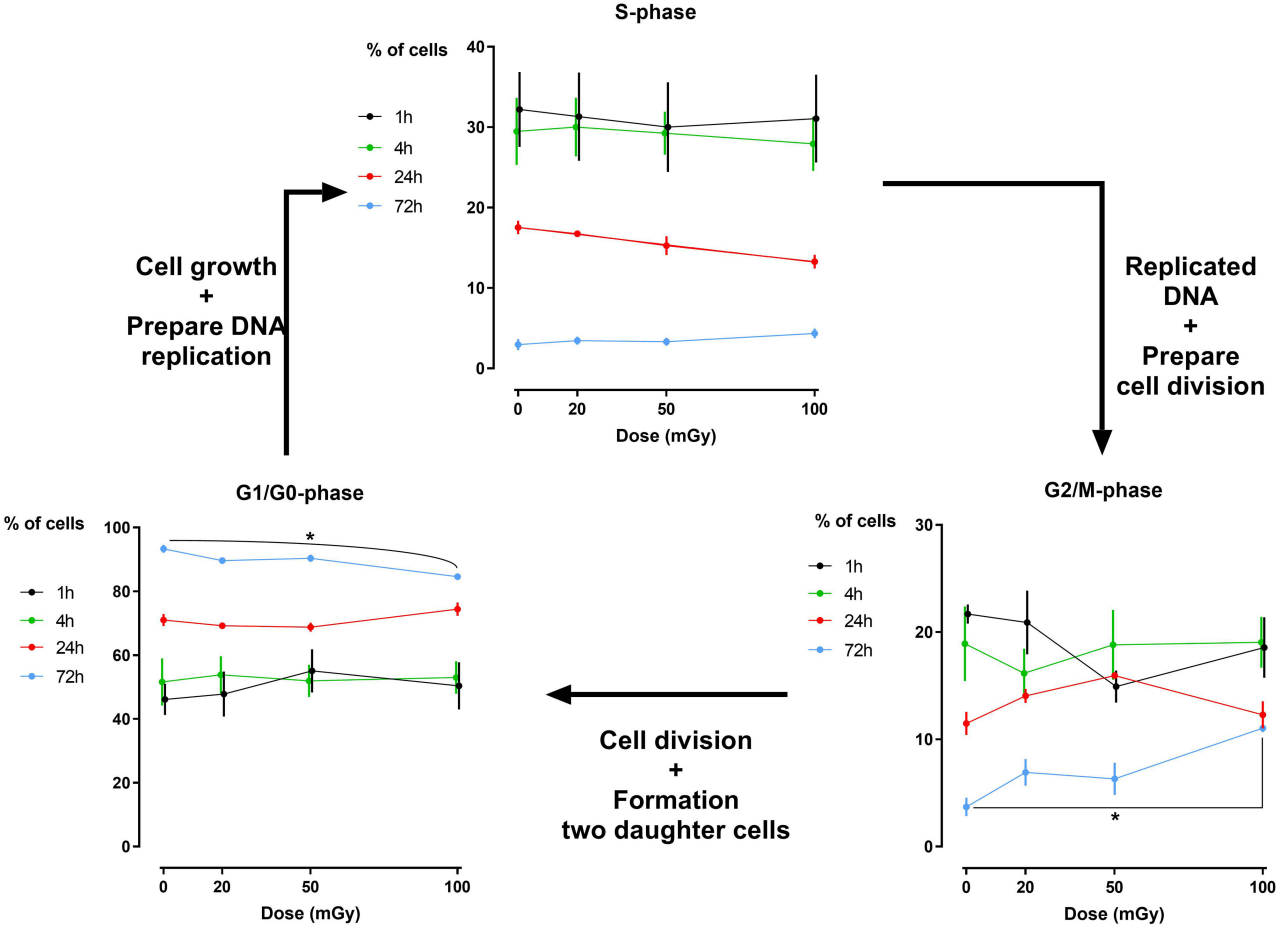
Cell cycle analysis of dental pulp stromal cells from deciduous teeth. Dental pulp stromal cells from deciduous teeth (SHEDs) show a significantly decreased number of G_1_/G_0_ phase cells 72 h following X-irradiation with 100 mGy. Coincidently, a significant increase in the number of G_2_/M phase cells was observed. *P ≤ 0.05.

Analysis of the percentage of cells that reside in a specific phase of the cell cycle has revealed that exposure to low doses of X-rays (< 100 mGy; 900 mGy/h) does not induce major cell cycle changes in dental stromal cells (SHEDs sand SCAPs) (*N* = 3 for each cell type), while a high dose of 2Gy of X-rays does (500 mGy/min; Supplementary Figure 1). Except for a slightly reduced number of G_1_/G_0_ phase cells 72 h after irradiation in SHED (*P* = 0.019) and a slight increase in G_2_/M phase cells 72 h after irradiation in SHED (*P* = 0.040) following a dose of 100 mGy, no changes were observed (Figure 2). We did observe that the amount of G_1_/G_0_ phase cells increases over time, whereas the amount of S- and G_2_/M phase cells decreases over time, with almost no more cells in the S-phase after 72 h. Positive controls after exposure to 2 Gy of X-rays can be found in Supplementary Figure 1.

### Low Dose X-irradiation Rapidly Decreases the Amount of Quiescent Cells

**Figure 3:**
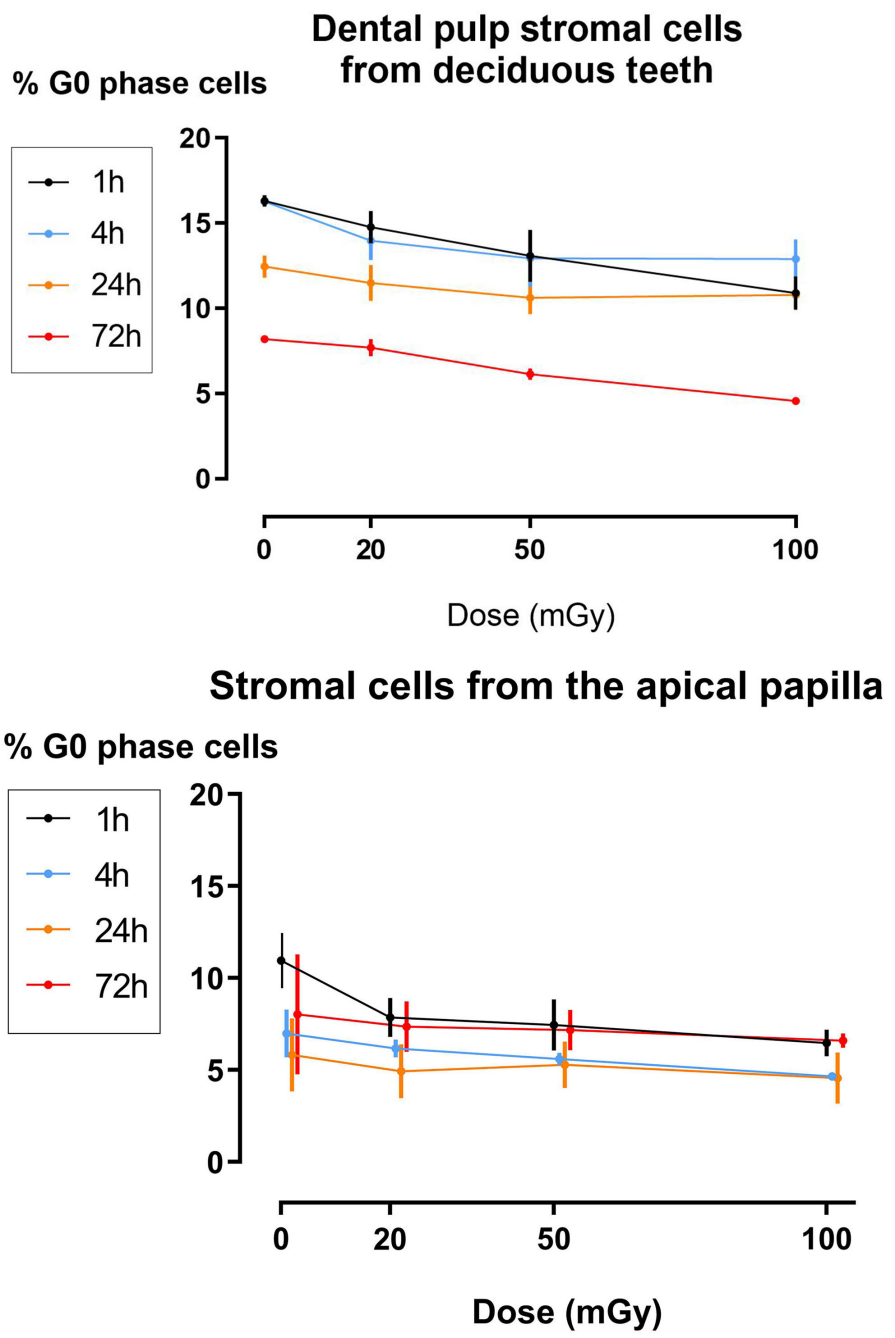
Dose response of the percentage of G_0_ phase dental pulp stromal cells from deciduous teeth and stromal cells from the apical papilla following low dose X-irradiation. The percentage of G_0_ phase cells is plotted against the time after X-irradiation. Significances are summarized in the Table 3.

The effect of exposure to low doses of X-rays on cellular quiescence, determined by measuring the percentage of G_0_ phase cells, was most pronounced 1 h after irradiation with 100 mGy. This was observed in SHEDs and SCAPs (*N* = 3). However, SHEDs showed still significant dose-dependent decreases in the percentage of quiescent cells 4 and 72 h after irradiation (Figure 3 and Table 3). In SCAPs, only a decrease was seen 1 h after irradiation with 100 mGy (*P* = 0.030). It was also observed that the number of G_0_ decreased significantly over time (Figure 3 and Table 3).

**Table 3:** Significant differences in the percentage of quiescent cells in dental stromal cells.

**Comparison**	**Dental pulp stromal cells from deciduous teeth (*P*-value)**	**Stromal cells from the apical papilla (*P*-value)**
1 h:CTRL vs. 50 mGy	0.0107	N.A.
1 h:CTRL vs. 100 mGy	<0.0001	0.0296
1 h:20 mGy vs. 100 mGy	0.0011	N.A.
4 h:CTRL vs. 50 mGy	0.0072	N.A.
4 h:CTRL vs. 100 mGy	0.0064	N.A.
72 h: CTRL vs. 100 mGy	0.0025	N.A.
72 h:20 mGy vs. 100 mGy	0.0145	N.A.

### Low Dose Radiation Does Not Induce Premature Senescence in Dental Mesenchymal Stromal Cells

**Figure 4:**
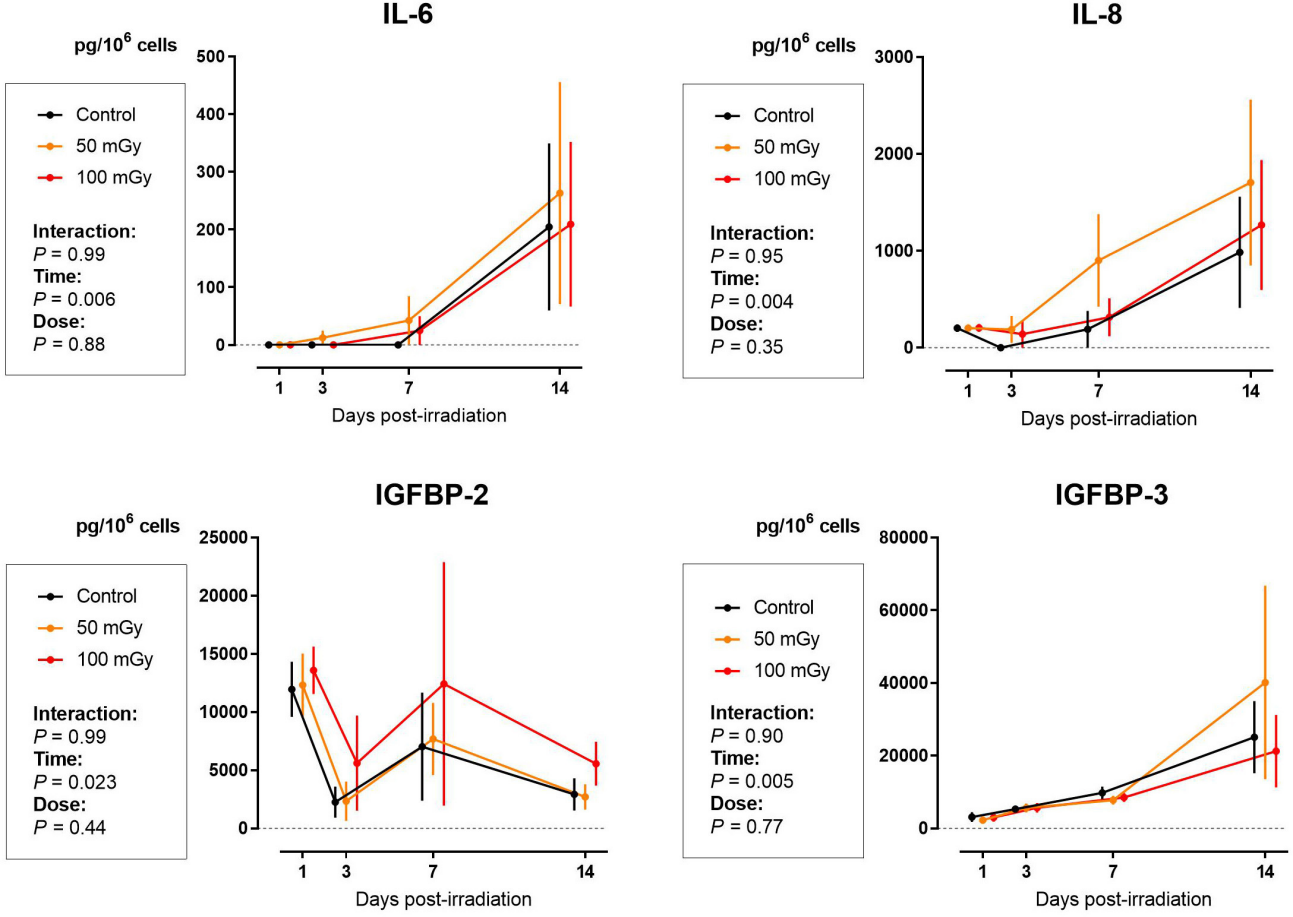
Senescence-associated secretory phenotype (SASP) proteins secretion in dental pulp stromal cells from deciduous teeth (SHEDs) following low dose ionizing radiation exposure. The amount of interleukins (IL)-6 and IL-8, as well as the levels of insulin-like growth factor binding proteins (IGFBP)-2 and IGFBP-3 are indicate as normalized by the amount of cells. Two-way analysis of variance shows that time after exposure is the major contributor to the observed effects (*P*_*time*_ = 0.023).

Enzyme-linked immunosorbent assay (ELISA) for SASP markers IL-6, IL-8, IGFBP-2, and IGFBP-3 showed no signs of radiation-induced premature cellular senescence in SHEDs, DFSCs, and SCAPs up to 14 days after exposure (*N* = 3 for each cell type). Although the values for IL-6 and IL-8 in SHEDs increased significantly 14 days after irradiation exposure, this was mostly due to the time in culture, rather than a radiation-induced effect (*P*_*time*_ = 0.006 and *P*_*time*_ = 0.004, respectively). Levels of IGFBP-2 in SHEDs showed changes over time, but overall there was a decreasing trend, which was not influenced by radiation dose (*P*_*time*_ = 0.022). Finally, in SHEDs, IGFBP-3 showed a time dependent increase (*P*_*time*_ = 0.005; Figure 4).

**Figure 5:**
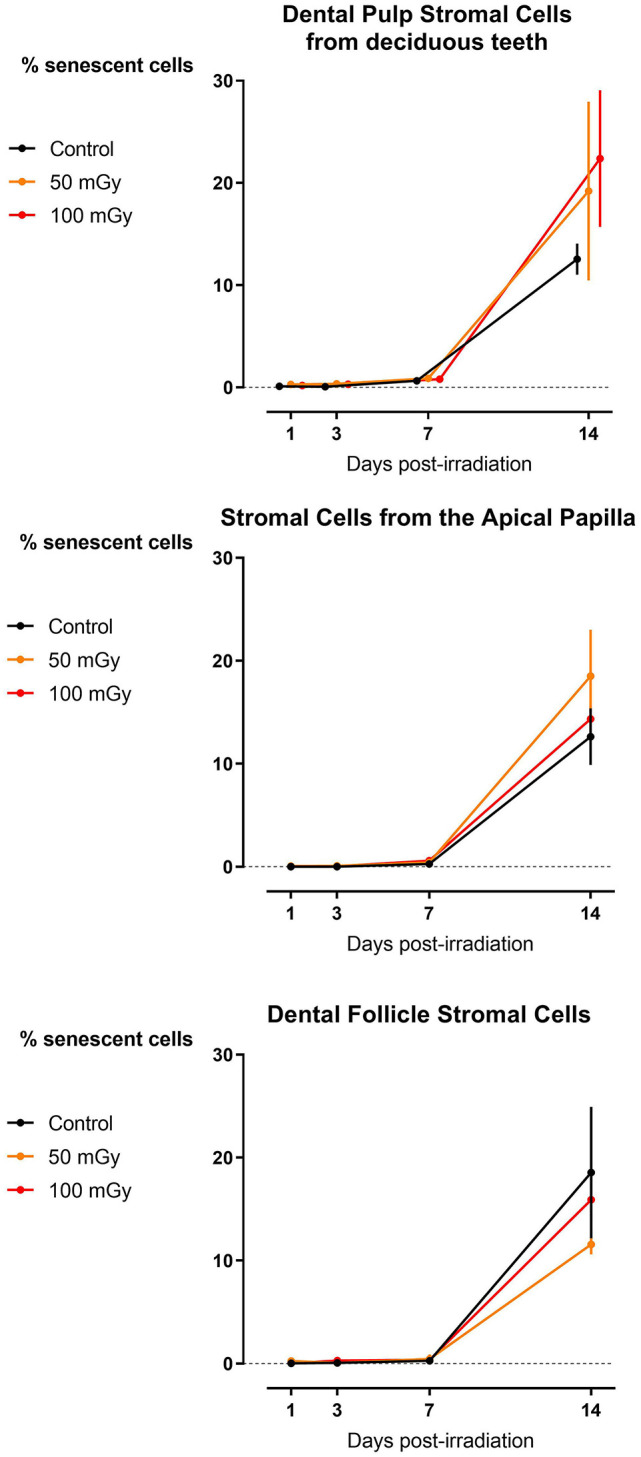
β-galactosidase assay in dental mesenchymal stromal cells. The percentage of senescent cells are indicated as normalized to the levels of the control samples at day 1 post-irradiation. Two-way analysis of variance shows that time after exposure is the major contributor to the observed effects (*P*_*time*_ < 0.0001 for all cell types).

The data from SASP markers were confirmed by the β-galactosidase assay (41). Data from dental stromal cells show that there is an increase in the percentage of senescent cells, but this increase is time-dependent. Low dose radiation exposure (<100 mGy; 900 mGy/h) does not induce cellular senescence in SHEDs, DFSCs, and SCAPs (*N* = 3 for each cell type; Figure 5).

## Discussion

Determining the biological effects of low dose IR exposure is currently the greatest challenge in radiation protection. We aimed to investigate the DDR and its consequences in human dental mesenchymal stromal cells (i.e., SHEDs, DFSCs, and SCAPs) after exposure to X-ray doses with the use of medical imaging beam settings (<100 mGy; 900 mGy/h). SHEDs, DFSCs, and SCAPs are dental mesenchymal stromal cells defined as non-clonal cultures of stromal cells containing stem cells with different multipotent properties, committed progenitors, and differentiated cells. MSCs support the maintenance of other cells, and the capacity of MSCs to differentiate into several cell types makes the cells unique and full of possibilities (65). Therefore, maintaining the genetic stability of MSCs is of paramount importance. MSCs can accumulate genotoxic damage following IR exposure, which is either repaired efficiently, or they can accumulate irreversible damage. This persisting damage could lead to malignant transformation of the stem cells (1).

The formation and repair kinetics of DNA DSBs was monitored via γH2AX/53BP1 immunostaining. Additionally, the impact of low dose radiation on cell cycle progression, cellular quiescence and premature cellular senescence were investigated. We report a significant increase in the amount of DNA DSBs 30 min and 1 h after low dose IR exposure (<100 mGy; 900 mGy/h). As γ-H2AX foci may not always be associated with DNA DSB, co-localization with repair proteins 53BP1 has thus been used to further optimize the sensitivity of DNA DSB quantification (66, 67). Repair kinetics clearly showed that the number of DSBs in dental stromal cells returned to baseline levels 24 h after IR exposure. Despite the DNA DSBs being repaired, there is a possibility that misrepair has occurred as a consequence of non-homologous end joining (68, 69). Furthermore, a slight G_2_/M phase arrest was seen 72 h after irradiation in SHEDs, but not in SCAPs or DFSCs. Next, IR exposure resulted in reduced levels of G_0_ cells in SHEDs and SCAP. However, in SCAP the decrease was only statistically significant 1 h after irradiation and only for irradiation with 100 mGy. For SHEDs, on the other hand, also 4 and 72 h after irradiation a statistically significant decrease was observed. Finally, low dose X-ray exposure (<100 mGy; 900 mGy/h) did not result in radiation-induced premature senescence in SHEDs, DFSCs, and SCAPs.

It is well-known that exposure to X-rays can induce DNA DSBs, which are considered very harmful because unrepaired/misrepaired DSBs could result in mutations, chromosome rearrangements/aberrations, and loss of genetic information (28, 66, 70, 71). Our results show that exposure to low dose IR with medical imaging beam settings (<100 mGy; 900 mGy/h) induces significant increases in the number of DNA DSBs in dental mesenchymal stromal cells 30–60 min after irradiation (72). Similar results have been reported in human mesenchymal stem cells before (3, 47, 73–77). However, some studies report a persistent increase of γH2AX foci up to 48 h after irradiation, which was not observed in our study (3, 73, 74). Linear regression analysis showed that the number of DNA DSBs increases linearly with the IR dose. The slopes in SHEDs, DFSCs and SCAPs ranged from 0.019 to 0.026 DNA DSBs per mGy. This is equivalent to 19–26 DNA DSBs per Gy, which is consistent with data published previously (24, 78–81).

The formed DNA DSBs did not affect cell cycle progression in SCAPs, but we did observe a slight G_2_/M phase arrest in SHEDs 72 h following 100 mGy exposure. Although this increase was minimal, it was statistically significant. This is in line with previous publications indicating that cells exhibit G_2_/M phase arrest following exposure to high IR doses (37–39, 47). However, there are data indicating that exposure to high doses of IR results in G_1_ arrest in mesenchymal stem cells (75). Furthermore, the lack of cell cycle changes in SCAPs is in line with data from Kurpinski et al., who also observed no changes in cell cycle distribution in bone marrow mesenchymal stem cells following X-irradiation with 100 mGy (82). Our data, taken together with data from literature, indicate that the effect of X-irradiation on cell cycle progression is cell type dependent.

Our cell cycle data reveal minimal changes in the G_1_/G_0_ phase of the cell cycle. However, our data show for the first time a significant decrease in the amount of quiescent or G_0_ phase cells in SHEDs 72 h after X-irradiation with 100 mGy (dose rate: 900 mGy/h). This would indicate that if the amount of G_1_/G_0_ phase remains constant, but the amount of G_0_ phase cells decreases, that the amount of G_1_ phase cells increase proportionally to the decrease of G_0_ phase cells. This indicates that low doses of IR stimulate SHEDs to re-enter the cell cycle. It has been described that certain extrinsic stresses such as IR-induced reactive oxygen species, which are generated by radiolysis of water following IR exposure, can stimulate stem cell to re-enter the cell cycle (83). This could, at least partly, explain our observations.

Finally, we did not observe radiation-induced cellular senescence following exposure to low doses of IR except for SHEDs where a slight increase in G_2_/M arrest was observed 72 h after irradiation with 100 mGy (dose rate: 900 mGy/h). However, our data clearly showed a time-dependent induction of senescence. This was seen both in results from the X-gal assay, which is considered the gold standard, as in analysis of the SASP. It has been reported before that high doses of IR can induce cellular senescence in mesenchymal stem cells (47–49, 51, 84). However, evidence of low dose IR-induced senescence is scarce (3, 85) and contradict our data. On the other hand, there are studies that support our findings (74, 86). Due to these contradicting data and the fact that low dose radiation-induced senescence is poorly investigated, it is impossible to conclude at this time whether low doses of IR do cause cellular senescence in these cell or not. More detailed studies on this matter are warranted (13).

In addition, future research from our study would benefit from the investigation of cell apoptosis and cell proliferation. Indeed, analysis of cell apoptosis would increase our understanding if after DNA damage the processes of cell death are triggered or not. Analysis of cell proliferation would confirm the results shown with the analysis of the cell cycle and could highlight a possible change in proliferation as a result of DNA damage. Other techniques for investigating cellular senescence, such as looking at different protein levels by Western Blotting, would clarify the relationship between senescence and cell cycle status after low dose IR. Differentiation potential after low dose IR exposure would also be an additional point to investigate. In conclusion, we found that exposure of dental mesenchymal stromal cells to low doses of X-rays with medical imaging beam settings (<100 mGy; 900 mGy/h) results in the induction of DNA DSBs and that the number of DNA DSBs increases linearly with the radiation dose. After 24 h, these DNA DSBs are efficiently repaired and returned to baseline levels. Yet, how these initial DNA DSBs affects long-term functionality of dental mesenchymal stromal cells is inconclusive. We report for the first time, to the best of our knowledge, that exposure to low IR doses results in an acute dose-dependent decrease in the number of quiescent SHEDs and SCAPs, which is still observed 72 h after irradiation after X-irradiation in SHEDs. However, we did not find adverse effects on cell cycle progression. No persistent cell cycle changes, nor induction of premature cellular senescence were observed. Although this is in line with previous studies, there are also studies indicating that low doses of IR, albeit with different beam qualities, can cause cell cycle arrest and senescence. Our data highlight the need for more detailed and extensive studies on the effects of exposure to low doses of IR as used in CBCTs.

## Data Availability Statement

The raw data supporting the conclusions of this article will be made available by the authors, without undue reservation.

## Ethics Statement

The studies involving human participants were reviewed and approved by Comité d'Evaluation de l'Ethique des projets de Reserche Biomédicale Paris Nord. Written informed consent to participate in this study was provided by the participants' legal guardian/next of kin.

## Author Contributions

NB was the principle author of the paper, conducted all experiments, and analyses described in this manuscript. LG aided NB in performing experiments and analyses described in the manuscript, and critically reviewed the manuscript. JW aided NB in performing experiments and analyses described in the manuscript, and critically reviewed the manuscript. RV aided NB in performing experiments described in the manuscript, and critically reviewed the manuscript. BS contributed to design of the experiments, revised the manuscript, and approved its publication. SB contributed to the conception of the study and to revision the manuscript and approved its publication. SL is the university co-director of NB, critically revised the manuscript, and approved its publication. RJ contributed to the conception, design of the experiments, lead of the DIMITRA study, critically revised the manuscript, and approved its publication. IL is the university director of NB, critically revised the manuscript and approved its publication. MM aided NB in performing analyses, the interpretation thereof, contributed to the conception, design of the experiments, responsible of the experiments that were performed, and critically revised the manuscript and approved its publication. BB helped with data interpretation, scientific guidance and preparation of the manuscript. All authors contributed to the article and approved the submitted version.

## Conflict of Interest

The authors declare that the research was conducted in the absence of any commercial or financial relationships that could be construed as a potential conflict of interest.

## Supplementary Material

Supplementary Figure 1 Cell cycle response of the dental stromal cells following 2 Gy X-irradiation. ^*^*P* ≤ 0.05, ^**^*P* ≤ 0.01, ^***^*P* ≤ 0.001, ^****^*P* ≤ 0.0001.
