# Supplementary figures and images for: In vitro Assessment of the DNA Damage Response in Dental Mesenchymal Stromal Cells Following Low Dose X-ray Exposure
Source: Front Public Health. 2021 Feb 15;9:584484. doi: 10.3389/fpubh.2021.584484 (PMC7939020; doi:10.3389/fpubh.2021.584484)

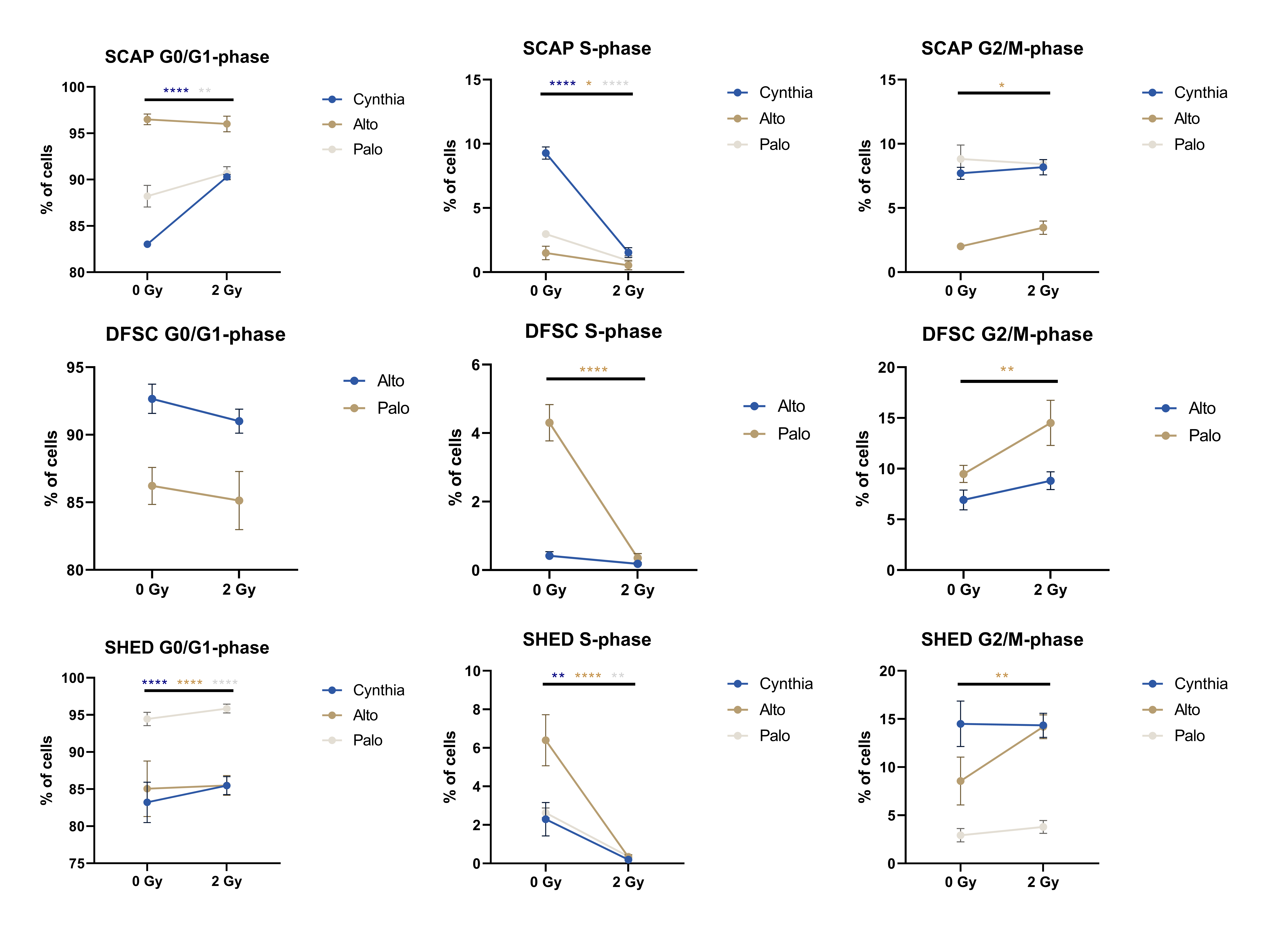

Supplement: Supplementary Figure 1 — Cell cycle response of the dental stromal cells following 2 Gy X-irradiation. *P ≤ 0.05, **P ≤ 0.01, ***P ≤ 0.001, ****P ≤ 0.0001. [file Image_1.TIF]
